# Disruption of mitochondrial homeostasis with artemisinin unravels anti-angiogenesis effects via auto-paracrine mechanisms

**DOI:** 10.7150/thno.33353

**Published:** 2019-09-17

**Authors:** Kuan-Hao Tsui, Meng-Yu Wu, Li-Te Lin, Zhi-Hong Wen, Yi-Han Li, Pei-Yi Chu, Chia-Jung Li

**Affiliations:** 1 Department of Obstetrics and Gynecology, Kaohsiung Veterans General Hospital, Kaohsiung, Taiwan;; 2 Department of Obstetrics and Gynecology, National Yang-Ming University School of Medicine, Taipei, Taiwan;; 3 Department of Pharmacy and Master Program, College of Pharmacy and Health Care, Tajen University, Pingtung County, Taiwan;; 4 Institute of Biomedical Sciences, National Sun Yat-sen University, Kaohsiung 80424, Taiwan;; 5 Department of Emergency Medicine, Taipei Tzu Chi Hospital, Buddhist Tzu Chi Medical Foundation, New Taipei, Taiwan;; 6 Department of Emergency Medicine, School of Medicine, Tzu Chi University, Hualien, Taiwan;; 7 Department of Biological Science, National Sun Yat-sen University, Kaohsiung, Taiwan;; 8 Department of Marine Biotechnology and Resources, National Sun Yat-sen University, Kaohsiung, Taiwan;; 9 Marine Biomedical Laboratory and Center for Translational Biopharmaceuticals, Department of Marine Biotechnology and Resources, National Sun Yat-sen University, Kaohsiun, Taiwan;; 10 Department of Pathology & Immunology, Baylor College of Medicine, Houston, Texas, USA;; 11 School of Medicine, College of Medicine, Fu Jen Catholic University, New Taipei city, Taiwan;; 12 Department of Pathology, Show Chwan Memorial Hospital, Changhua, Taiwan; 13 Department of Health Food, Chung Chou University of Science and Technology, Changhua, Taiwan

**Keywords:** artemisinin, mitochondria, VEGF, angiogenesis, auto-paracrine

## Abstract

**Rationale**: Tumor angiogenesis promotes tumor development, progression, growth, and metastasis. Metronomic chemotherapy involves the frequent administration of low-dose chemotherapeutic agents to block angiogenic activity and reduce side effects.

**Methods**: MDA-MB-231 cells were treated with various concentrations of artemisinin (ART) and vinorelbine (NVB) and the cytotoxic effects of ART/NVB were determined using the CCK-8 assay. Mitochondrial reactive oxygen species (ROS) levels, mitochondrial membrane potential (∆Ψm) and mass were assessed using MitoSOX, TMRE and MitoTracker green staining. Western blot analysis was used to quantify the expression of autophagy-related proteins. Herein, by using bioinformatics analysis and experimental verification, we identified CREB as a master in MDA-MB-231 cells.

**Results**: We found that artemisinin (ART), which exhibits anti-angiogenic and anti-cancer effects via mitochondrial regulation, synergized with vinorelbine (NVB) to inhibit MDA-MB-231 cell proliferation. ART and NVB cooperated to regulate mitochondrial biogenesis. CREB acted as a crucial regulator of PGC1α and VEGF, which played critical roles in NVB-dependent growth factor depletion. Moreover, CREB suppression significantly reversed mitochondrial dysfunction following ART/NVB co-treatment. In addition, combination treatment with ART and NVB significantly suppressed tumor growth in a nude mouse xenograft model, with downregulated CREB and PGC1α expression levels observed in tumor biopsies, in agreement with our *in vitro* and *ex vivo* data.

**Conclusions**: These findings support the hypothesis that ART affects cancer and endothelial cells by targeting the auto-paracrine effects of VEGF to suppress mitochondrial biogenesis, angiogenesis, and migration between cancer cells and endothelial cells.

## Introduction

Angiogenesis is essential for tumor growth and metastasis, and controlling tumor-associated angiogenesis is a promising tactic for limiting cancer progression 1. Anti-angiogenic strategies to treat cancers have been designed to target the vascular endothelial growth factor (VEGF), in order to inhibit neovascularization. Vinorelbine (NVB) is a semi-synthetic vinca alkaloid that is used clinically to block angiogenesis and suppress tumor growth 2, 3. However, anti-angiogenic therapies may exert both autocrine and paracrine VEGF responses within tumors. Direct tumor cell stimulation by VEGF may protect cells from apoptosis and increase their resistance to conventional chemotherapy and radiotherapy 4. Therefore, anti-VEGF treatments used in combination with conventional chemotherapy and radiotherapy should dramatically improve treatment for cancer patients 5.

Mitochondria are the hotspots of current cancer research, including the signal transmission between the nucleus and the mitochondria, and how the mitochondria control the metabolism of cancer cells, and then the anti-cancer treatment targeting mitochondria. In addition, mitochondrial metabolism and biogenesis are key regulators of endothelial cell function during angiogenesis 6. Interestingly, VEGF stimulated the angiogenesis by promoting the mitochondrial functions and biogenesis 7. The biogenesis in tumor cells is regulated by multiple transcription factors. The cAMP response element-binding protein (CREB) is a member of the leucine-zipper family of DNA-binding proteins that is highly expressed in various cancers, such as lung cancer, glioblastoma, breast cancer, melanoma, and diffuse malignant mesothelioma 8-11. CREB was previously found to play key biogenic roles by regulating PGC1α, NRF1, ERRα, and Sirt3 activation 12. A key activator of mitochondrial biogenesis in cancer is PGC1α, a transcription factor that regulates fatty acid oxidation, oxidative phosphorylation, metabolism, and apoptosis. However, elevated mitochondrial biogenesis due to oncogenesis increases the cellular biosynthetic and respiratory capacity by upregulating mitochondrial metabolism to stimulate rapid proliferation and tumor progression 13, 14. Hence, targeting mitochondria can be a good strategy for killing cancer cells.

Artemisinin (ART) is the active component of the plant *Artemisia annua* L. that has been used in traditional Chinese medicine to treat fevers 15 and is approved worldwide for treating and preventing malaria 16. ART has been used traditionally for anti-malarial and anti-viral therapy for several years 17, 18. ART potentiates the activities of other chemotherapeutic agents and, therefore, may be useful for treating cancer as a monotherapy, or in combination with other cytotoxic drugs 19, 20. In addition, combination therapy has gained much attention in experimental research. Thus, ART cannot only inhibit tumor growth directly *in vitro* and *in vivo*, but can also enhance the anti-tumor effects of cytotoxic agents and chemotherapeutic agents 21, 22. ART potentiates the activities of other chemotherapeutic agents and therefore may be useful, not only as a monotherapy, but also in combination with other cytotoxic drugs for cancer treatment 23, 24.

ART can induce cell death through a mitochondrial pathway 25, 26. However, the therapeutic effects of ART and NVB on mechanisms associated with mitochondrial homeostasis have not been explored in breast cancer. Here, we examined the ability of chemotherapy combined with ART to affect CREB-mediated autocrine and paracrine interactions between breast tumor cells and vascular endothelial cells. Our findings suggest that mitochondrial metabolism is a promising target for combination ART/NVB therapy in tumor microenvironments.

## Materials and Methods

### Cells, cell culture, and reagents

Two human breast carcinoma cell lines (MDA-MB-231 and MCF-7) were purchased from the Bioresource Collection and Research Center (Hsinchu, Taiwan). The human mammary gland epithelial cell line (MCF-10A) and human umbilical vein vascular endothelial cell (HUVECs) line were purchased from the American Type Culture Collection (VA, USA). HUVECs were grown under standard conditions in endothelial cell growth medium supplemented with the EGM-2 Bullet Kit. HUVECs at passages 4-8 were used for all experiments. The cancer cells were grown in F-12K medium containing 10% fetal bovine serum. Cells were maintained at 37ºC in a humidified atmosphere with 5% CO_2_. Artemisinin and vinorelbine were purchased from Sigma Chemical Co. (MO, USA).

### Determination of the combination index (CI) and dose-reduction index

The interaction between ART and NVB was quantified by determining the CI, as described 27, 28.

### Cell-viability assay

Cell-viability assays were performed as described 29.

### Mitochondrial functional analysis

Cells were harvested after various treatments, washed, resuspended in culture medium, and stained with MitoTracker green (10 nM), MitoSOX (5 μM) and TMRM (500 nM) (Molecular Probes) at 37°C for 30 min. The cells were washed and analyzed by flow cytometry (FACSCalibur, BD Bioscience). ATP levels were determined by measuring the absorbance at 450 nm using a colorimetric assay kit (Abcam) and a microplate reader.

### Reverse transcriptase-polymerase chain reaction (RT-PCR) and PCR array

An RT^2^ Profiler Custom PCR Array (Qiagen) was used to examine the mRNA levels of 84 genes. GAPDH mRNA expression was detected as a reference gene. Several negative controls were included in each run. Total RNA was extracted with REzol (Protech Technology). mRNA-expression levels were analyzed in SYBR Green-based quantitative real-time PCR (qRT-PCR) assays (StepOne; Applied Biosystems), with β-actin and GAPDH serving as reference genes. Relevant primer sequences are shown in Table S1.

### Western blot analysis

A Western blot immunoassay was performed as described in a previous report 30.

### Chromatin immunoprecipitation (ChIP) assays

ChIP assays were performed as described 31. Briefly, the CREB protein was fixed with DNA by incubation with 1% formaldehyde for 10 min at room temperature (RT). Cells were harvested and sonicated to fragment the DNA. CREB protein-DNA complexes were precipitated using an anti-CREB antibody (Abcam) and Dynabeads Protein G (ThermoFisher Scientific). Potential binding sites were amplified using the primers shown in Supplemental Table S1.

### Immunoprecipitation

Immunoprecipitation was performed with the total lysate of cells using a Dynabeads Protein G Kit (Novex, ThermoFisher Scientific). Briefly, 5 μg primary antibody (Table S2) was incubated with Dynabeads for 10 min at RT, and the resulting mixture was incubated with 500 μg total protein lysate for 10 min at RT. The beads were washed with PBST (phosphate-buffered saline containing 0.02% Tween-20) and heated to 70ºC for 10 min. The tube was then placed on a magnet, and the supernatant was loaded onto a gel.

### Isolation of nuclear and mitochondrial proteins

Nuclear and mitochondrial protein isolation was performed as described 32, 33.

### Small-interfering RNA (siRNA) transfections

For siRNA transfections, MDA-MB-231 cells were transfected with 100 pmol siRNAs using Lipofectamine 3000 and analyzed 3 days post-transfection. CREB siRNAs were purchased from Cell Signaling Technology.

### Metabolic Assays

All metabolic assays were performed with Biovision (Mountain View, CA) metabolism kits. Specifically, citrate (K655), succinate (K649), fumarate (K633), malate (K637), alpha-ketoglutarate (K677) and 2-hydroxyglutarate (K213) were used in accordance to manufacturer specifications.

### Preparation of conditioned medium from MDA-MB-231 cells

HUVECs were grown to subcon fluence and treated for 24 h with serum-free medium to obtain the corresponding cm. HUVEC cells were treated with CM collected from MDA-MB-231 cells for 24 h. The medium was then collected, filtered to remove cell debris, and stored at -20°C until for later experiments. The *in vitro* angiogenesis experiments were evaluated by mixing conditioned medium of cancer cells with HUVECs culture medium (serum free) in a 1:1 mixture.

### Cell migration and invasion assay

The migration and invasion assay was performed as described 34.

### 5-ethynyl-2′-deoxyuridine (EdU) cell-proliferation assays

Cell-proliferation assays were conducted by labeling genomic DNA with EdU (a DNA precursor molecule that is incorporated during DNA replication) and measuring such incorporation using the Click-iT^TM^ EdU Flow Cytometry Assay Kit (Invitrogen, CA, USA) 35.

### Angiogenesis assays

Endothelial tube formation, Matrigel plug, aortic ring, and chick chorioallantoic membrane assays were performed as described 36.

### *In vivo* solid tumor xenografts assay

Specific pathogen-free male BALB/c nude mice were obtained from the National Laboratory Animal Center (Taipei, Taiwan). Mice were maintained in cages at 25ºC ± 2ºC on a 12-h light/dark cycle, with free access to food and water. For solid tumor xenografts, MDA-MB-231 breast tumor cells (5 × 10^6^/100 μl PBS) were injected subcutaneously into the hind flank of SCID mice and grown for 7 days. Once the mice developed detectable tumors, ART and/or NVB treatment was initiated for 2 weeks. Tumor sizes were measured with calipers, and tumor volumes were calculated as the larger diameter (mm) × small diameter (mm)^2^/2. Tumor tissues excised from the back of sacrificed animals were used for Western blot analysis. Tumor tissues were also homogenized in Drabkin's reagent (Sigma Co.) and centrifuged (12,000 rpm, 10 min), after which the hemoglobin content of the supernatants were measured by determining the absorbance at 540 nm. All animal studies were handled in accordance with procedures approved by the Institutional Animal Care and Use Committee of Chang Bing Show Chwan Memorial Hospital.

### Immunofluorescence and immunohistochemistry

MDA-MB-231 cells were treated with ART and NVB for 24 h, washed with PBS, fixed with 4% paraformaldehyde in PBS, and permeabilized with 0.2% Triton X-100 on ice for 5 min. After washing with PBS twice, the cells were incubated in blocking solution for 30 min and then incubated overnight at 4°C with appropriate antibodies. The cells were washed with PBS and incubated for 60 min with a FITC-conjugated goat anti-mouse secondary antibody at a 1:200 dilution. Subsequently, the cells were counterstained with DAPI (Sigma Co.) in PBS for 20 min and images were taken with a TCS-SP2 confocal laser-scanning microscope (Leica Inc., Germany). Tumors were paraffin-embedded and treated with specific antibodies for immunohistochemical (IHC) evaluation. Tumor tissue IHC was performed as described 37. Images were acquired with confocal microscope (C1-Si, Nikon) with a 60× oil objective. All confocal images were analyzed using TissueQuest software (TissueGnostics).

### Evaluation of biochemical parameters

Serum biochemical parameters were assessed as described 30. Briefly, the levels serum alanine aminotransferase (ALT), aspartate aminotransferase (AST), blood urea nitrogen (BUN), and creatinine (Cr) were used to assess changes in liver and renal functions. These biochemical parameters were analyzed using a Hitachi 717 Chemistry Analyzer (Hitachi, Tokyo, Japan).

### Statistical analysis

Data presented are the mean ± standard error of the mean (S.E.M.) from at least 3 independent experiments and were analyzed using a Student's t-test with a two-tailed distribution between groups. All calculations were performed using GraphPad Prism, 6.01. All quantification was performed using ImageJ (NIH) and MicroP software 33. CI values of < 1, = 1, and > 1 indicated synergism, additive effects, and antagonism, respectively.

## Results

### ART sensitized breast cancer cells to chemotherapeutic agents

**Figure 1:**
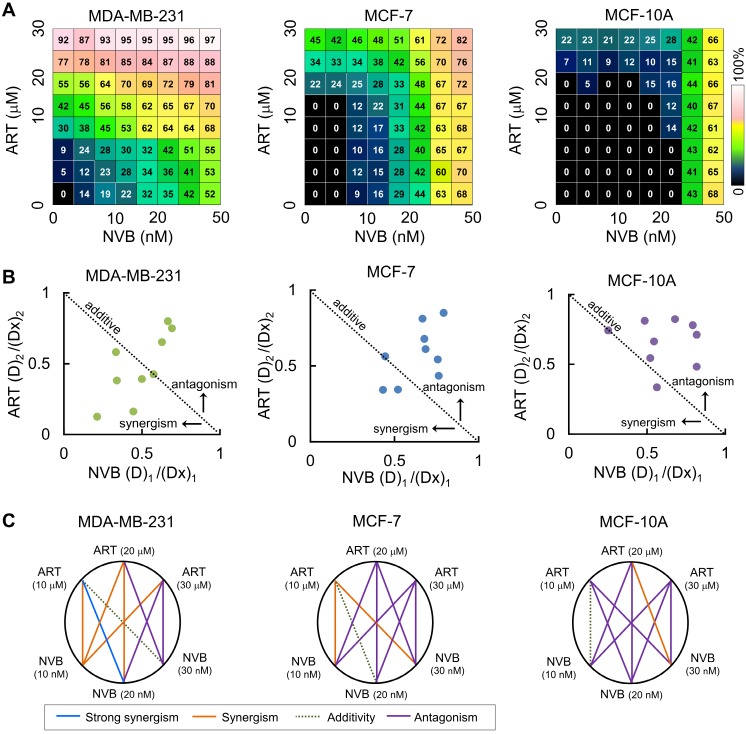
ART synergized with NVB to inhibit breast cancer cell line proliferation. (A) An 8 × 8 dose matrix was generated for ART × NVB. The effects of combined ART/NVB treatment on MDA-MB-231, MCF-7, and MCF-10A cell proliferation. (B) Synergy was also described by preparing isobolograms to compare the doses needed to reach 50% inhibition along an equal-effect contour to those along a predicted contour, based on a model of dose additivity. A CI of 1.0 (dotted line) reflects additive effects, whereas values greater than or less than 1.0 indicate antagonism or synergy, respectively. All dosing combinations show synergy as determined by the Chou-Talalay method.

Combination studies of low-dose ART with NVB have not been reported for breast cancer cells; therefore, we tested whether ART could synergize with NVB leading to enhanced suppression of cell viability. Strong synergy was observed between ART and NVB in MDA-MB-231 cells, but not in MCF-7 cells (Figure 1A). In the drug screen from which this combination was selected, we found evidence of synergy in MDA-MB-231 cells for 39 out of 64 drug combinations that suppressed cell proliferation, 19 of which showed mild levels of selectivity over MCF-7. In addition, neither ART nor NVB changed the growth of MCF-10A cells. These results showed that including ART at a sub-toxic concentration inhibited MDA-MB-231 cell growth more than NVB treatment alone, without increasing cytotoxicity in MCF-10A cells. ART and NVB co-treatment caused synergistic cytotoxicity as revealed by an isobologram: most data points were below the line of additive effects for MDA-MB-231 cells (Figure 1B). The results for MDA-MB-231 cells showed a highly synergistic effect, with all CI values < 1. Figure 1C shows a normalized isobologram for various tested values in MDA-MB-231, MCF-7, and MCF-10A cells. CI values < 1 at the tested doses indicated synergy, and the tested doses showed improvement over either drug alone. The combination of 10 μM ART and 20 nM NVB showed the best CI score in MDA-MB-231 cells (Figure 1C).

### Imbalance of mitochondrial dynamics in MDA-MB-231 cells after ART/NVB exposure

**Figure 2:**
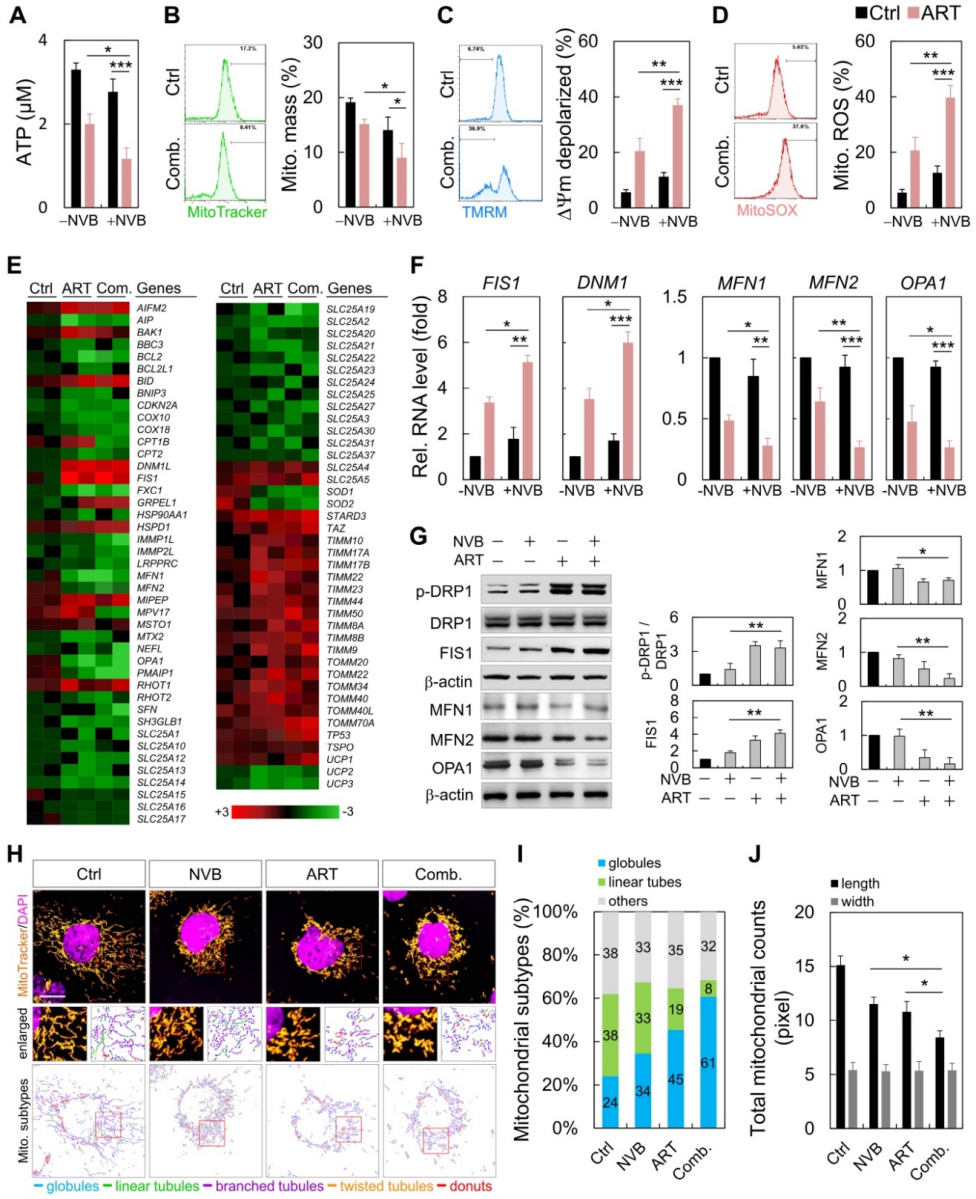
ART/NVB treatment regulated mitochondrial functions and dynamics in MDA-MB-231 cells. (A) ATP levels, (B) mitochondrial mass, (C) mitochondrial membrane potential, and (D) mitochondrial ROS levels in MDA-MB-231 cells treated with ART or/and NVB. (E) Heatmap demonstrating the relative expression levels of mitochondrial genes, based on PCR array analysis. (F, G) mRNA- and protein-expression levels were verified by qRT-PCR and immunoblotting. Protein expression levels were normalized to β-actin. (H) Mitochondria in MDA-MB-231 cells, with or without ART/VNB treatment, were labeled with MitoTracker and subjected to confocal microscopy. The mitochondria were classified using MicroP software according to their morphology: globules, linear tubules, branched tubules, twisted tubules, and donuts. (I) Three major types of mitochondria were quantified: globules, linear tubes, and others. (J) The total length and width of each mitochondrion was determined. *P < 0.05, **P < 0.01, and ***P < 0.001. Scare bar = 20 μm

MDA-MB-231 cells were treated with ART or/and NVB for 24 h, which resulted in decreases in the mitochondrial mass, ATP and reactive oxygen species (ROS) levels, and membrane potential (Figure 2A-D). We employed an 84-probe mitochondrial PCR microarray to analyze the transcriptional profile of ART/NVB-treated MDA-MB-231 cells. We focused on the expression levels of genes related to mitochondrial dynamics, several of which were changed by ≥2-fold (Figure 2E). The PCR array results were verified using specific primers that were available for *FIS1, DNM1, MFN1, MFN2*, and *OPA1*. These expression results were verified by qRT-PCR and Western blotting in MDA-MB-231 cells treated with ART/NVB, which revealed significant changes after drug treatment (Figure 2F, G). Fluorescence images confirmed the distribution of mitochondria. The mitochondria were classified into five distinct types according to their morphological characteristics: globules, linear tubules, branched tubules, twisted tubules, and donuts (Figure 2H). Compared with the Ctrl group, the mitochondria of the combined-treatment group increased mitochondrial fragmentation from 24% to 61%, while the tubular mitochondria decreased from 38% to 8% (Figure 2I). The average length of mitochondria in ART/NVB-treated cells significantly decreased compared to that in untreated cells. The mitochondrial widths did not differ following ART/BVB treatment (Figure 2J).

### Regulation of the CREB/PGC1α transcription factor axis by ART/NVB-induced changes in mitochondrial quality

**Figure 3:**
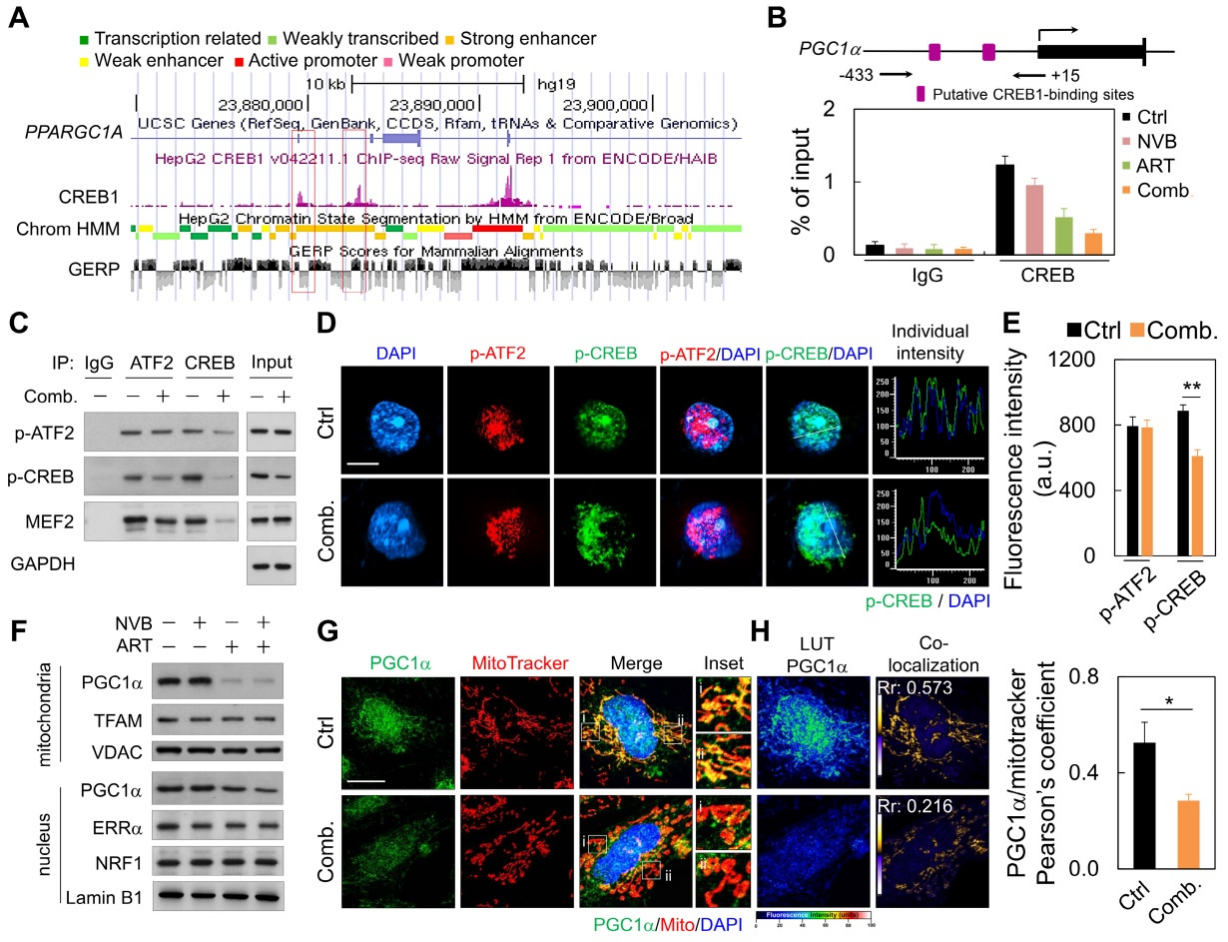
ART/NVB decreased PGC1α expression by inhibiting the ATF2/CREB pathway. (A) Scheme (UCSC Genome Browser hg19) of the human genomic region encompassing the PGC1α promoter. The purple peaks represent the CREB-binding regions, according to ENCODE. The red square shows the amplified region in the PGC1α promoter. (B) ChIP assay performed with MDA-MB-231 cells treated with ART or/and NVB. The PGC1α promotor region was immunoprecipitated with an anti-CREB antibody and amplified by qRT-PCR. (C) Immunoblot analysis of proteins immunoprecipitated from ART/NVB-treated cells using antibodies against IgG, ATF2, or CREB. (D) Representative immunofluorescence patterns of p-ATF2 (Thr69-71) and p-CREB (Ser133). (E) Quantification of Pearson's co-localization coefficients between Pa-TF2 (Thr69-71) and p-CREB (Ser133). (F) Relative mitochondrial and nuclear levels of proteins related to mitochondrial biogenesis, detected in lysates from ART/NVB-treated breast cancer cells by Western blotting. (G) Confocal images and mitochondrial co-localization analysis of PGC1α. The insets highlight two representative co-localization images (30× magnification of the regions in the white squares). (H) Calculation and quantification of Pearson's co-localization coefficients between PGC1α and MitoTracker in ART/NVB-treated cells. *P < 0.05 and **P < 0.01. Scare bar = 10 μm

We next explored upstream regulators that potentially mediate the decreased mitochondrial protein expression. ART/NVB treatment might regulate the activity of PGC1α, which is a key effector of mitochondrial biogenesis 38. Publicly available ChIP and sequencing data showed that CREB can bind directly to the PGC1α promoter (Figure 3A). ChIP-PCR with MDA-MB-231 cells showed that endogenous CREB bound to PGC1α under normal conditions, whereas treatment with ART or ART/NVB markedly abrogated CREB binding to PGC1α (Figure 3B). Since MEF2 and ATF2 have been reported to act as co-activators with CREB to drive PGC1α transcription 38, we tested whether ART/NVB interacted with other transcriptional factors of ATF2 in MDA-MB-231 cells. ATF2 was immunoprecipitated in control cells, whereas less p-CREB was precipitated in ART/NVB-treated cells (Figure 3C and Figure S2). Consistently, less p-ATF2, p-CREB, and MEF2 were immunoprecipitated with an anti-CREB antibody in ART/NVB-treated cells. Immunofluorescence also revealed that ART/NVB caused p-CREB inactivation and showed an irregular non-spherical and broken staining pattern, in contrast to p-ATF2 immunofluorescence (Figure 3D). ART/NVB treatment significantly decreased the fluorescence intensity of p-CREB (Figure 3E).

The effects of ART and/or NVB treatment on PGC1α subcellular localization were detected by immunoblotting and immunofluorescence. ART/NVB-stimulated MDA-MB-231 cells showed decreased mitochondrial PGC1α levels. ART/NVB significantly suppressed nuclear expression of the PGC1α protein, but not the ERRα and NRF1 proteins (Figure 3F and Figure S2). Immunofluorescence microscopy revealed that PGC1α co-localized with mitochondria in control cells, although the co-localization was particularly attenuated along the edges of ART/NVB treated cells (Figure 3G). Moreover, control cells showed co-localization of PGC1α and MitoTracker. ART/NVB treatment significantly decrease the overlap coefficient between PGC1α and mitochondria. These co-localizations also decreased in ART/NVB-treated cells (Figure 3H).

### Loss of CREB impacted mitochondrial function and tumor progression

**Figure 4:**
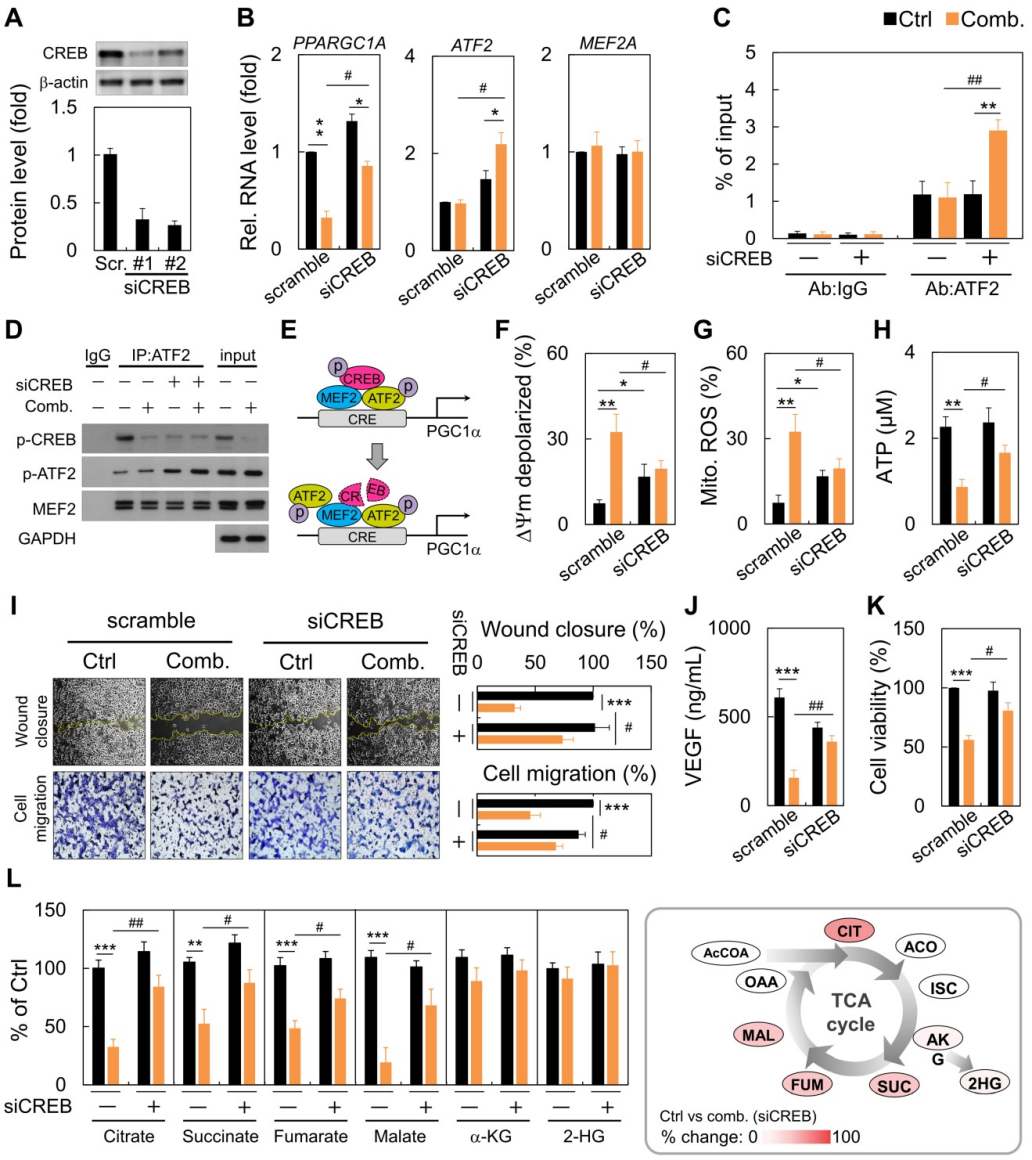
Reversal of the synergistic effects of combination ART/NVB treatment by down-regulating CREB expression in MDA-MB-231 cells. (A) Immunoblotting and quantification of CREB protein levels in MDA-MB-231 cells after treatment with siCREB. The lower panel shows densitometric quantification of bands relative to those in the scrambled siRNA group. (B)* PPARGC1A, ATF2*, and *MEF2A* mRNA levels in cells treated with ART/NVB or vehicle control and either siCREB or a scrambled siRNA control. (C) ChIP assay results with MDA-MB-231 cells following transfection with siRNAs (siCREB or scrambled siRNA) and treatment with ART/NVB or vehicle control. Potential ATF2-binding sites in the *PGC1α* locus were amplified by qRT-PCR. (D) SDS-PAGE and immunoblot analysis of proteins immunoprecipitated with anti-ATF2 and IgG antibodies in MDA-MB-231 cells transfected with siRNAs (siCREB or scrambled siRNA) and treatment with ART/NVB (or vehicle control). (E) Proposed model whereby ART/NVB regulates interactions between CREB, ATF2, and MEF2. (F-H) Mitochondrial membrane potential, and mitochondrial ROS and ATP production in CREB-knockdown cells treated with ART/NVB. (I) Motility and migration were examined in CREB-knockdown cells after ART/NVB treatment. (J) VEGF levels in CREB-knockdown cells after ART/NVB treatment. (K) MDA-MB-231 cells were treated with ART/NVB for 24 h after transfection with siCREB, and cell viability was determined by performing CCK8 assays. (L) Measurement of TCA cycle intermediates in cancer cells silenced for CREB and exposed to ART/NVB for 24 h. Quantification analysis of TCA cycle metabolites was relative to their respective control. * *P* < 0.05, ** P < 0.01, *** P < 0.001, as compared with each control. #*P* < 0.05 and #*P* < 0.01, as compared with scrambled-siRNA group

Next, we performed RNA-interference experiments against CREB. RNA interference reduced CREB expression by almost 70% (Figure 4A). Following CREB down-regulation, ART/NVB mildly inhibited PGC1α expression. However, ART/NVB treatment increased ATF2 expression, but not MEF2 expression, in CREB-knockdown cells (Figure 4B). By ChIP-qPCR, we further demonstrated robust recruitment of ATF2 to the PGC1α promoter versus the IgG control (Figure 4C). CREB knockdown under physiological conditions reduced basal ATF2 expression by ~three-fold. We confirmed that ATF2 bound PGC1α in siCREB cells by co-IP, using an anti-ATF2 antibody. Although siCREB did not affect MEF2 expression in ART/NVB-treated cells, it dramatically elevated ART/NVB-mediated ATF2 phosphorylation (Figure 4D and Figure S2). These results indicate that ART/NVB-specific inhibition of CREB and knockdown of CREB expression by siRNAs elicited a compensatory increase in ATF2-binding activity to drive PGC1α expression in ART/NVB-treated cells (Figure 4E). Moreover, siCREB attenuated ART/NVB-mediated mitochondrial dysfunction, as reflected by a higher membrane potential, and lower mitochondrial ROS and ATP generation in the siCREB group. CREB knockdown restored mitochondrial functions (Figure 4F-H). siCREB elevated wound closure and cell migration in ART/NVB-treated cells, and increased the VEGF concentration and cancer cell survival (Figure 4I-K). Comparative analysis revealed a significant increase of citrate, succinate, fumarate and malate content in siCREB conditioned samples (Figure 4L), whereas α-ketoglutarate (α-KG) and 2-hydroxyglutarate (2-HG) levels were unaffected by ART/NVB exposure.

### ART/NVB-treated cells (cmART/NVB) controlled endothelial cell motility and *in vitro* and *ex vivo* angiogenesis through paracrine mechanisms

**Figure 5:**
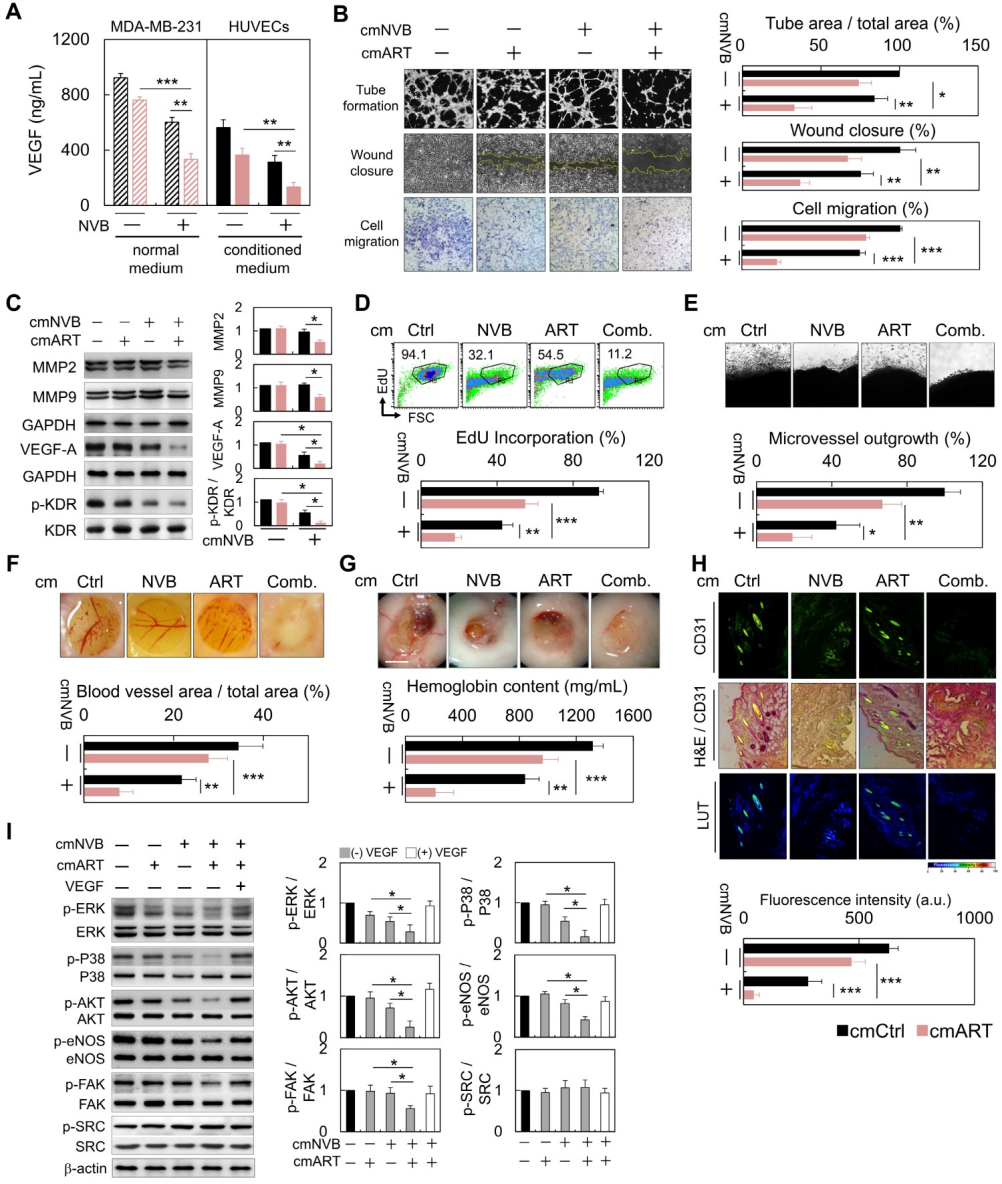
Anti-angiogenesis and anti-proliferation functions of cm. from cancer cells on HUVECs through paracrine stimulation. (A) VEGF levels in ART/NVB treated cancer cells and cmART/NVB treated HUVECs (B) Quantification of the effects of cm. from MDA-MB-231 cells on angiogenesis by performing capillary-like tube-formation, wound-healing, and migration assays. HUVECs were cultured with cellular VEGF protein derived from a cell lysate and with VEGF protein secreted into cm. by cancer cells treated with or without ART or/and NVB. (C) Western blot analysis of MMP2, MMP9, VEGFA, and p-KDR expression in HUVECs after cmART/NVB exposure. (D) These cell populations were subsequently treated with cmART/NVB. Cellular DNA synthesis was measured by EdU-incorporation assays and flow cytometry. The percentage of S phase cells is indicated. (E) Images showing a rat aorta ring incubated for 7 days with the indicated cm. Representative images of a rat aortic ring sprouting in response to the indicated treatments. Quantitative sprout growth data are shown. Scare bar = 200 μm. (F) The chorioallantoic membrane of a 7-day-old chick embryo onto which a single small ring was placed. These rings were treated with cm. from Ctrl or ART/NVB-treated cells for 48 h. (G) Five-week-old SCID mice were injected with 0.5 mL of Matrigel mixed with cmART/NVB. Matrigel was injected as the vehicle control. After 14 days, the skin of the sacrificed experimental animals was pulled back to expose the intact Matrigel plugs, which were analyzed. Scare bar = 0.5 mm. (H) Inhibition of blood vessel formation by cmART/NVB. The Matrigel plugs were fixed, sectioned, and stained with an anti-CD31 antibody and counterstained with hematoxylin and eosin (H&E; magnification, ×200). (I) Western blot analysis of the relative protein-expression levels of VEGF and downstream signaling molecules following cmART/NVB treatment in HUVECs. The cellular expression levels observed in the absence of drug treatment were normalized as 1.0. *P < 0.05, **P < 0.01, and ***P < 0.001

To investigate whether ART/NVB-mediated MDA-MB-231 cell-derived VEGF regulates angiogenesis, MDA-MB-231 cells were treated with conditioned medium (cm). Cm. not only reduced the level of VEGF (Figure 5A), but also inhibited tube formation, wound closure and migration in HUVECs (Figure 5B). Cancer cell invasion is mediated by breakdown of the basement membrane, a process dependent on extracellular matrix degradation by the matrix metalloproteinase (MMP) enzymes MMP-2 and MMP-9. Western blot analysis suggested that MMP-2 and MMP-9 expression decreased in the cmART/NVB-treated HUVECs cells. (Figure 5C). No apparent cytotoxic effects were observed in the experiments when endothelial cells were treated with ART/NVB (Figure S1). Taken together, these observations indicate that VEGF played a paracrine role in modulating vascular permeability associated with breast cancer after ART/NVB treatment.

### VEGF derived from conditioned medium from

Treating HUVECs with conditioned medium from cancer cells decreased the proportion of cells incorporating EdU (Figure 5D). We further examined the effect of cmART/NVB on vessel sprout formation using the rat aorta ring and chick embryo chorioallantoic membrane assay. Fewer vessel sprouts formed in the presence of cmART/NVB (Figure 5E, F). In contrast to cmCtrl-treated Matrigel plugs that induced prominent vascularization, plugs loaded with cmART/NVB remained relatively inert (Figure 5G). Angiogenesis was evaluated by measuring expression of the blood vessel marker CD31 in the Matrigel plugs. At 7 days post-implantation, the plugs derived from cmCtrl stimulation alone displayed strong CD31 expression (Figure 5H). Significant inhibition of angiogenesis was found in the plugs mixed with ART/NVB, with reduced CD31 levels. Next, we examined whether ART/NVB-induced expression of VEGF regulates KDR phosphorylation in endothelial cells through a paracrine loop. MDA-MB-231 cells were treated with cm. alone or cm. containing exogenous VEGF, and incubated with endothelial cells. VEGF promotes angiogenesis by activating multiple signal-transduction pathways involving SRC/FAK, MAPK, or AKT/eNOS signaling, or NO production, which activate VEGF receptor 2 (i.e., KDR). The ERK and p38 MAPK pathways mediate the proliferative and migratory activities of VEGF in endothelial cells, and the AKT/eNOS/NO pathway mediates VEGF-induced endothelial survival and proliferation 39. However, SRC and FAK activation are also important for endothelial cell migration in response to VEGF. cmART/NVB effectively inhibited VEGF-induced angiogenic pathways involving ERK and p38 MAPK activation and the AKT/eNOS/NO pathway, but not SRC activation (Figure 5I).

### Anti-angiogenic and anti-tumor effects of ART/NVB: *in vivo* assay

**Figure 6:**
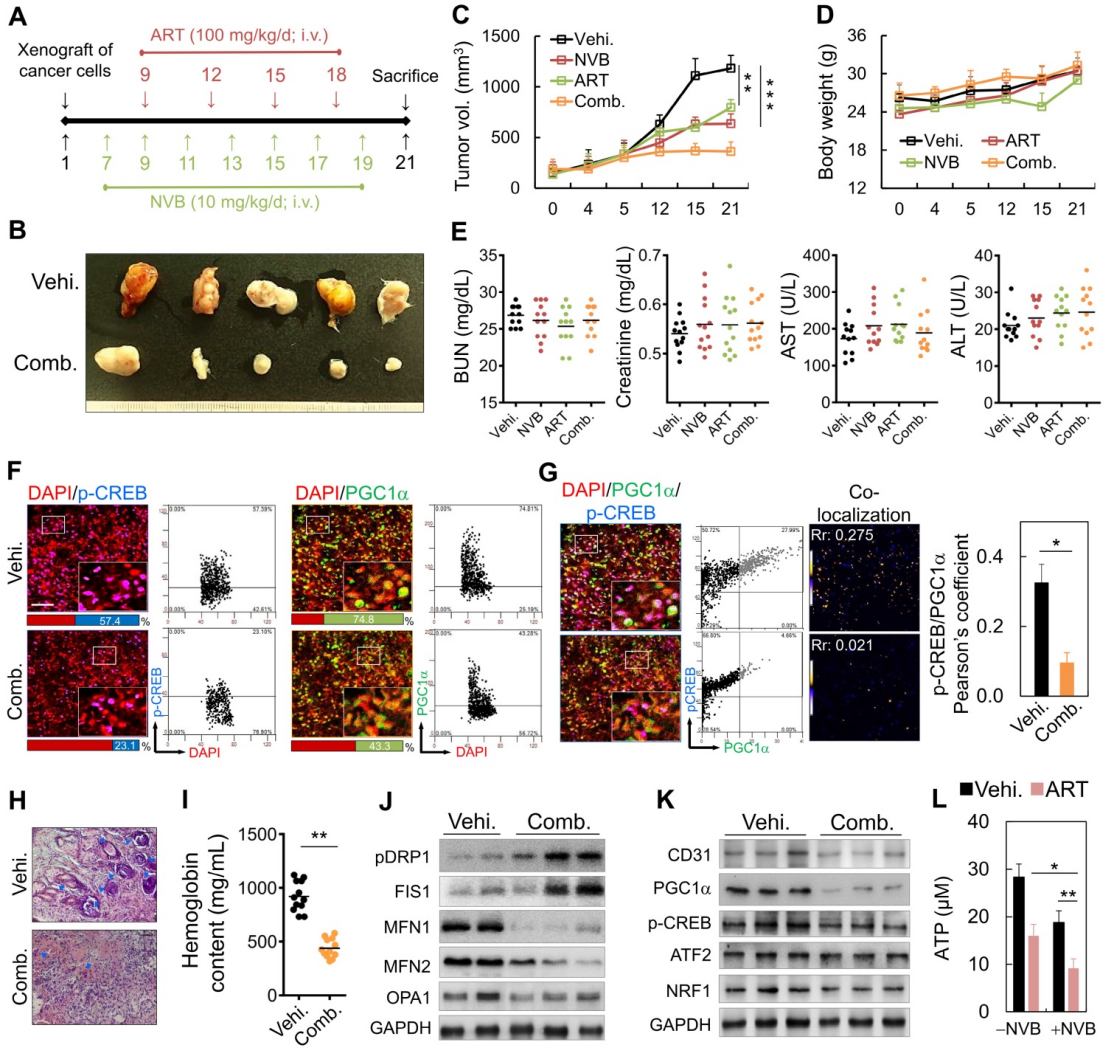
Metronomic low-dose ART chemotherapy combined with NVB exhibited anti-angiogenic and anti-tumor activities *in vivo*. (A) Flow chart for the* in vivo* experimental design and treatment schedule. (B) Volumes of tumors in mice treated with ART combined with NVB. Mice were treated by i.v. injection with vehicle or ART/NVB. (C, D) Quantification of tumor volumes and body weights in mice treated with NVB and ART/NVB. (E) Blood biochemical analysis indicated that no significant changes occurred in the serum ALT, AST, BUN, or Cr levels. ALT, alanine aminotransferase; AST, aspartate aminotransferase; BUN, blood urea nitrogen. (F) Tumor sections were observed by immunostaining for CREB and PGC1α. Quantitative analysis of CREB- and PGC1α-positive cells in tumors treated with ART and NVB. The positive cells showed fluorescence in tumors treated with NVB and ART/NVB. Confocal microscopy images of co-staining and scatter plots for pC-REB, PGC1α, and DAPI are shown. Representative dot plots showing CREB and PGC1α expression in mouse biopsies, obtained using a flow cytometry-like analysis system (TissueQuest). (G) Confocal and co-localization analyses of p-CREB and PGC1α. CREB and PGC1α colocalization is presented as the product of the differences from the mean. Yellow or blue color pixels indicate colocalization or segregation, respectively. (H) Inhibition of blood vessel formation by ART/NVB treatment. A solid tumor was fixed, sectioned, and stained with H&E. Blue arrows indicate positive blood vessels. (I) Relative angiogenesis was analyzed based on the red blood cell hemoglobin level, as determined using the Drabkin method. (J, K) The protein-expression levels of CD31, pPGC1α, p-CREB, ATF2, NRF1, pDRP1, FIS1, MFN1, MFN2, and OPA1 were detected by western blotting of tumor tissue extracts. (L) ATP levels in tumor tissue extracts. *P < 0.05, **P < 0.01, and ***P < 0.001. Scare bar = 50 μm

We also examined the effects of low-dose metronomic chemotherapy in combination with ART on MDA-MB-231 cell growth in SCID mice (Figure 6A). On day 7 post-implantation, the mice were administered a metronomic dose, which significantly inhibited tumor growth (Figure 6B). Tumor volumes decreased significantly after intravenous (i.v.) injections of ART/NVB for 14 days, relative to those in mice administered the vehicle control (Figure 6C). Low-dose metronomic NVB chemotherapy combined with ART did not show toxicity or cause weight loss during the 21-day treatment period (Figure 6D). Serum ALT, AST, creatinine, and BUN levels between the ART/NVB-treated and vehicle-treated groups also showed no significant differences (Figure 6E). We also examined CREB and PGC1α protein expression levels in tumor biopsies obtained from tumor-bearing mice after ART/NVB treatment. Tumors from ART/NVB-treated mice contained fewer CREB- and PGC1α-positive cells than those from NVB-treated mice (Figure 6F), and ART/NVB treatment significantly decreased the Pearson coefficient between CREB and PGC1α (Figure 6G). The microvessel density in the low-dose NVB metronomic chemotherapy plus ART group was much lower than that of the NVB group. Angiogenesis was quantified by measuring the hemoglobin contents in the tumor tissues, which were substantially lower in the combined treatment group than in the vehicle group (Figure 6H, I). The protein expression of CD31, PGC1α, CREB, and NRF1 decreased significantly in ART/NVB-treated mice versus NVB-treated mice (Figure 6J and Figure S2), in agreement with the mitochondrial dynamics-related expression data (Figure 2G). Thus, ART/NVB may have down-regulated PGC1α, p-CREB, and ATP levels *in vivo*, similar to the *in vitro* observations (Figure 6K, L and Figure S2).

## Discussion

Patients with breast cancer are commonly treated with vinorelbine, paclitaxel, irinotecan, or related chemotherapeutic agents 40, 41. Despite the relative chemosensitivity of breast cancer, the response rates to most commonly used cytotoxic agents were often lower than expected 42. Drug resistance remains one of the greatest challenges to effective chemotherapy, and there are no definitive strategies for treating chemoresistant tumors 41. Using a chemotherapeutic agent in combination with a molecularly targeted compound might achieve a better response rate than the chemotherapeutic agent alone 43, 44. Acquired drug resistance was partly mediated by the ability of tumor cells to circumvent and inhibit apoptosis 45, 46. Thus, new approaches with different anti-tumor mechanisms are being explored to overcome the limited effectiveness of current treatment modalities for breast cancer.

We discovered that ART enhances NVB-suppressed VEGF expression and that it potentially regulates cell motility *in vitro*, which ultimately can translate to the modulation of tumor angiogenesis *in vivo*. Several lines of evidence support this hypothesis. Specifically, CREB and PGC1α played important roles in ART/NVB-dependent regulation of mitochondrial functions during human breast tumor progression. Moreover, CREB expression was involved in drug sensitivity to ART And ART/NVB blocked VEGF from interacting with the tumor cell surface receptor KDR and stimulated VEGF/KDR-mediated tumor cell motility, in an autocrine manner. cmART/NVB inhibited VEGF-dependent down-regulation of KDR phosphorylation, resulting in suppressed endothelial cell motility and capillary-like tubule formation in endothelial cells, in a paracrine manner. ART/NVB-dependent VEGF suppression led to decreased breast tumor growth in a xenograft mouse model, and blocking tumor-derived VEGF significantly suppressed breast tumor progression and angiogenesis. Analysis of solid tumor specimens showed a significant correlation between CREB and PGC1α inactivation.

Combination therapy plays an essential role in the clinical treatment of malignant tumors 47. Targeting tumor blood vessel growth can suppress neo-angiogenesis and temporarily normalize tumor vessel structures, which may enhance the sensitivity of tumors to chemotherapy 48, 49. Combination therapy may also reduce drug resistance and side effects, as a given tumor is less likely to be resistant to multiple drugs simultaneously. Previous research demonstrated that intrinsic or constitutive activation of CREB and PGC1α may be critical for developing drug resistance and activating survival signals that counteract apoptosis in cancer cells. CREB and PGC1α inactivation, therefore, may represent a promising opportunity for widening therapeutic windows 50, 51. Here, we performed EdU assays and found ART inhibited the growth and proliferation of NVB-treated MDA-MB-231 cells in a dose-dependent manner, in agreement with previous reports describing the use of other chemotherapeutic drugs and cancer cells 22. Furthermore, our data indicated that ART exerted synergistic effects with NVB in suppressing the transcriptional activity of CREB and PGC1α, and inhibiting VEGF mRNA expression. Thus, inhibition of CREB and PGC1α by combination ART/NVB treatment may contribute profoundly to the increased sensitivity of cancer cells to combination chemotherapeutics. In agreement, several combinations of therapeutic treatments have enhanced cancer cell susceptibility by coordinating the inhibition of CREB and PGC1α 52, 53.

Targeting mitochondrial energy metabolism is a novel approach in cancer research and can be traced back to the description of the Warburg effect 54, 55. Mitochondria are complex organelles that affect the initiation, growth, survival and metastasis of cancer, and many aspects of mitochondrial biology beyond energy production actively contribute to tumorigenesis, such as biogenesis, ROS, metabolites, mitochondrial dynamics 56. Mitochondria undergo a process of continuous division and fusion, which is mainly regulated by two opposite processes: fission and fusion, which are regulated by DRP1, FIS1 and MFN1/2, OPA1, respectively. Imbalances in mitochondrial fission and fusion cause intracellular regulatory disorders that ultimately promote protooncogene activation 57. However, hypoxia is one of the most common features of the microenvironment in solid tumors and a major stimulator of tumor metastasis. Studies have shown that under normoxia, DRP1-mediated mitochondrial division is required for tumor cell metastasis 58. In a hypoxic microenvironment, DRP1-mediated mitochondrial fission not only enhances the metastatic activity of metastatic breast cancer cells MDA-MB-231, but also enhances the sensitivity of tumor cells to chemotherapeutic drugs 59. Therefore, mitochondrial homeostasis may serve as a potential therapeutic target for inhibiting breast cancer metastasis and increasing the effects of chemotherapy drugs.

The *in vivo* anti-angiogenic activity of metronomic low-dose NVB chemotherapy combined with ART was also demonstrated with tumor xenografts in SCID mice. Together, these data merit further investigation of the use of combined therapy as a potential cancer treatment strategy. At least two activities of ART/NVB treatment may contribute to its anti-angiogenic effect in tumors. Firstly, ART/NVB not only indirectly inhibited the migration, motility, and tube formation of HUVECs, but also directly inhibited the migration and motility of MDA-MB-231 cells. Secondly, ART/NVB treatment inhibited VEGF secretion by cancer cells. MMPs play important roles in cancer cell migration and invasion. MMP down-modulation in endothelial cells can suppress endothelial cell migration and invasion 60. Our data showed that ART/NVB treatment inhibit MMP-2 and MMP-9 expression in MDA-MB-231 cells. Angiogenic factors including VEGF and its receptor promote angiogenesis by activating multiple signal pathways, such as the MEK/ERK pathway (for endothelial cell proliferation), the PI3K/AKT/eNOS pathway (for endothelial cell survival), and the FAK/SRC/P38 MAPK signaling system (for endothelial cell migration) 61, 62. Our data showed that ART/NVB treatment decreased angiogenesis without inducing VEGF expression; thus, combined therapy may regulate angiogenic signaling pathways through direct and indirect mechanisms. Indeed, we confirmed that cmART/NVB activated multiple angiogenic signaling cascades, such as MEK/ERK-, AKT/eNOS/FAK-, and p38 MAPK-dependent pathways, which are essential for the inactivation of endothelial cells induced by angiogenic factors. However, our results also demonstrated that inhibition of endothelial cell migration and proliferation by cmART/NVB was not associated with SRC activation. These results indicate that cmART/NVB directly suppressed angiogenesis by inactivating intracellular angiogenic signal mediators without elevating VEGF expression.

We discovered that ART/NVB-mediated VEGF down-modulation decreased the proliferation and motility of breast tumor cells and HUVECs, in both autocrine and paracrine manners. Proliferation and motility could be activated by exogenous VEGF, indicating that a ligand/receptor (VEGF/KDR)-mediated signaling loop was responsible for the proliferative response. These findings suggest that VEGF and KDR are important biological markers for breast cancer malignancy and progression. ART/NVB effectively inhibited the autocrine and paracrine effects of VEGF by targeting both the tumor vasculature and breast cancer cells, thereby suppressing the proliferation, migration, angiogenesis, and growth of breast cancer tumors.

## Conclusions

**Figure 7:**
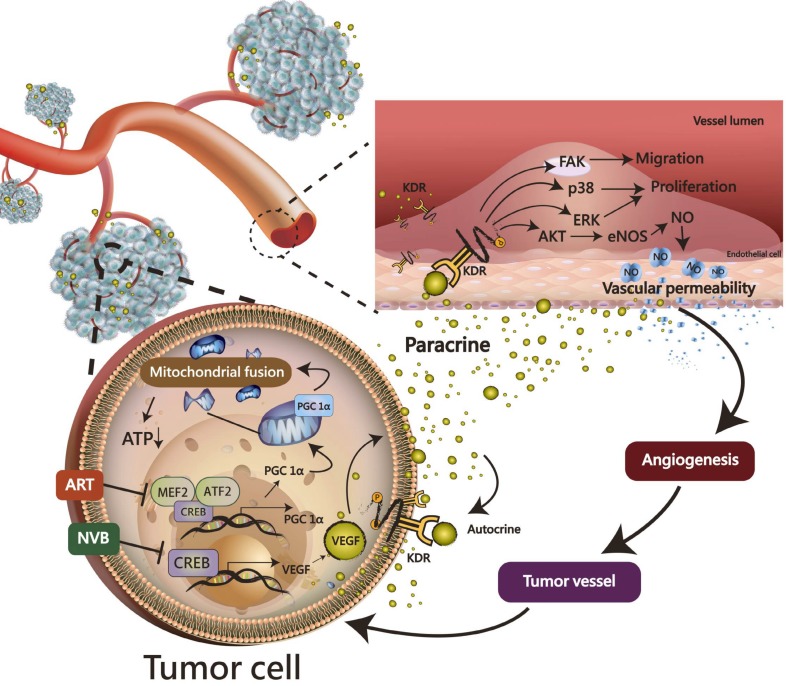
A working model of the effects of ART and NVB in both tumor cells and endothelial cells. The possible regulatory mechanism whereby ART/NVB treatment inhibits CREB- and PGC1α-mediated VEGF expression to block tumor angiogenesis through autocrine and paracrine mechanisms.

ART synergistically enhanced the NVB-mediated inhibition of mitochondrial biogenesis in MDA-MB-231 cells. Most importantly, the CREB-PGC1α signaling axis was involved in NVB sensitivity and the synergistic effects of ART/NVB co-treatment. Moreover, *ex vivo* and *in vivo* studies revealed that ART combined with NVB significantly suppressed tumor angiogenesis and tumor growth in a SCID mouse xenograft model, and changes in CREB and PGC1α expression in tumor tissues further demonstrated the molecular mechanisms *in vitro*. Our results warrant further investigation into the mechanism found in the xenograft mouse model, as a clear understanding of such a mechanism may facilitate the development of novel therapeutic approaches to suppress ART/NVB-regulated CREB and PGC1α-dependent VEGF expression, thereby controlling tumor growth and angiogenesis through autocrine and paracrine mechanisms (Figure 7).

## Supplementary Material

Supplementary figures and tables.

## Abbreviations

ART: artemisinin
ChIP: chromatin immunoprecipitation
CI: combination index
co-IP: co-immunoprecipitation
CREB: cAMP response element-binding protein
IHC: immunohistochemical
NVB: vinorelbine
VEGF: vascular endothelial growth factor
