# Supplementary material for: Disruption of mitochondrial homeostasis with artemisinin unravels anti-angiogenesis effects via auto-paracrine mechanisms
Source: Theranostics. 2019 Sep 17;9(22):6631–45. doi: 10.7150/thno.33353 (PMC6771251; doi:10.7150/thno.33353)
Supplement: Supplementary file 1 — Supplementary figures and tables. [file thnov09p6631s1.pdf]

Supplementary Table 1. Primer sequences designed for RT-PCR and ChIP

**Primers for RT-qPCR**	**sequences**
human MFN1-F	5'-GAGGTGCTATCTCGGAGACAC-3'
human MFN1-R	5'-GCCAATCCCACTAGGGAGAAC-3'
human MFN2-F	5'-CACATGGAGCGTTGTACCAG-3'
human MFN2-R	5'-TTGAGCACCTCCTTAGCAGAC-3'
human OPA1-F	5'-TGTGAGGTCTGCCAGTCTTTA-3'
human OPA1-R	5'-TGTCCTTAATTGGGGTCGTTG-3'
human DRP1-F	5'-ACCCGGAGACCTCTCATTCT-3'
human DRP1-R	5'-TGACAACGTTGGGTGAAAAA-3'
human FIS1-F	5'-GATGACATCCGTAAAGGCATCG-3'
human FIS1-R	5'-AGAAGACGTAATCCCGCTGTT-3'
human GAPDH-F	5'-TGCACCACCAACTGCTTAGC-3'
human GAPDH-R	5'-GGCATGGACTGTGGTCATGAG-3'

**Primer for ChIP**	**sequences**
PGC1α	5’-GCTGATCCACTTCTCATCCA<br>3’-GGACTGACTGGGACAAATT

Supplementary Table 2. Primary antibodies for immunoblotting, immunofluorescence and IP

Antigen	Host	Cat.	Type	Source
DRP1	Mouse	ab56788	monoclonal	Abcam
DRP1 Ser616	Rabbit	3455	monoclonal	Cellsignailing
FIS1	Rabbit	GTX111010	polyclonal	Genetex
MFN1	Rabbit	GTX133351	polyclonal	Genetex
MFN2	Mouse	ab56889	monoclonal	Abcam
OPA1	Rabbit	GTX129917	polyclonal	Genetex
ATF2	Mouse	ab47476	polyclonal	Abcam
ATF2 Thr71	Rabbit	ab32019	polyclonal	Abcam
CREB	Rabbit	ab32515	monoclonal	Abcam
CREB Ser133	Rabbit	ab32096	monoclonal	Abcam
MEF2	Rabbit	GTX101163	polyclonal	Genetex
PGC1α	Rabbit	ab54481	polyclonal	Abcam
TFAM	Rabbit	GTX103231	polyclonal	Genetex
ERRα	Rabbit	ab76228	monoclonal	Abcam
NRF1	Rabbit	ab175932	monoclonal	Abcam
VDAC	Rabbit	GTX114187	polyclonal	Genetex
CD31	Mouse	GTX20218	monoclonal	Genetex
ERK	Rabbit	GTX59618	polyclonal	Genetex
ERK Thr202	Rabbit	4511	polyclonal	Cellsignailing
P38	Rabbit	GTX110720	polyclonal	Genetex
P38 Thr180	Rabbit	GTX133460	polyclonal	Genetex
AKT	Rabbit	9272	polyclonal	Cellsignailing
AKT Ser473	Rabbit	9271	polyclonal	Cellsignailing
eNOS	Rabbit	GTX129843	polyclonal	Genetex
eNOS Ser1177	Rabbit	GTX129058	polyclonal	Genetex
FAK	Rabbit	ab40794	polyclonal	Abcam
FAK Tyr925	Rabbit	GTX129840	polyclonal	Genetex
SRC	Rabbit	ab47405	polyclonal	Abcam
SRC Tyr416	Rabbit	GTX133473	polyclonal	Genetex
MMP2	Rabbit	GTX104577	polyclonal	Genetex
MMP9	Rabbit	GTX100458	polyclonal	Genetex
VEGFA	Rabbit	Ab52917	monoclonal	Abcam
VEGFR2	Rabbit	9698	polyclonal	Cellsignailing
VEGFR2 Tyr1175	Rabbit	3370	polyclonal	Cellsignailing

Supplementary figure 1

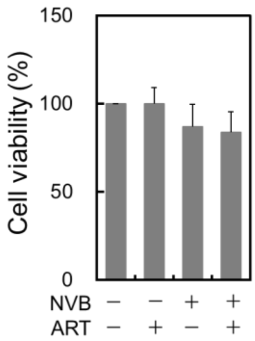

HUVEC cells were exposed to NVB/ART. Combined treatment resulted in significant growth inhibition of HUVEC cells, more than that by either compound alone; the degree of cytotoxicity in endothelial cells was not significant.

Supplementary figure 2

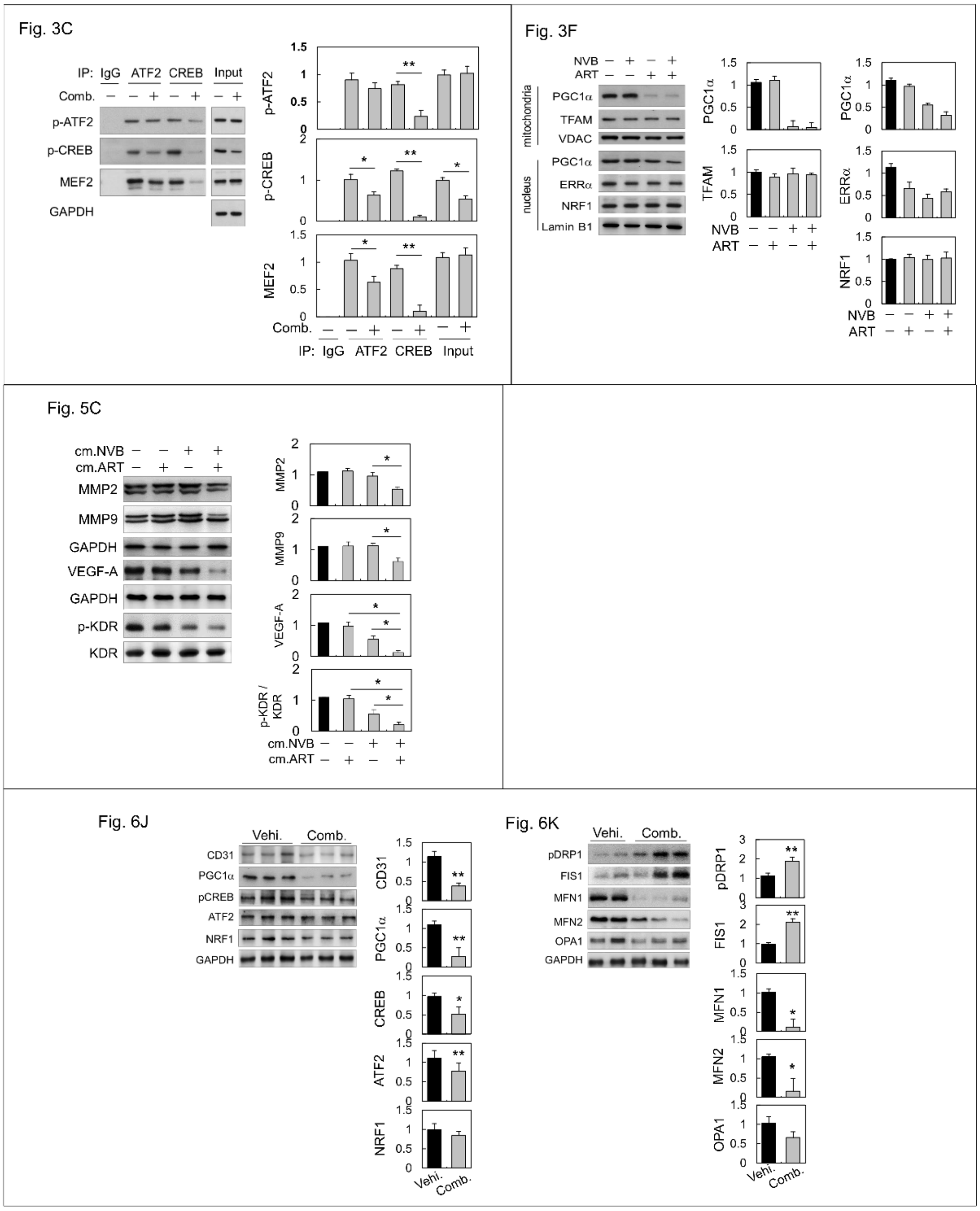

Protein-expression levels were verified by immunoblotting. Protein-expression levels were normalized to loading control.
